# Gonadotoxic Effects of Nilotinib in Chronic Myeloid Leukemia Treatment Dose in a Mouse Model

**DOI:** 10.4274/tjh.2016.0092

**Published:** 2017-06-01

**Authors:** Güldane Cengiz Seval, Sinan Özkavukçu, Murat Seval, Meltem Aylı

**Affiliations:** 1 Ufuk University Faculty of Medicine, Department of Hematology, Ankara, Turkey; 2 Ankara University Faculty of Medicine, Department of Obstetrics and Gynecology, Center of Assisted Reproduction, Ankara, Turkey; 3 Ankara University Faculty of Medicine, Department of Obstetrics and Gynecology, Ankara, Turkey

**Keywords:** Chronic myeloid leukemia, Nilotinib, Fertility, Mouse, gonads

## Abstract

**Objective:**

Tyrosine kinase inhibitors may have deleterious effects on spermatogenesis or folliculogenesis, resulting in male or female subfertility. The aim of this study is to determine the effect of nilotinib, which is used routinely to treat chronic myeloid leukemia, on spermatogenesis and folliculogenesis by using histopathological parameters.

**Materials and Methods:**

Ten male and ten female mice were orally treated with nilotinib at 20 mg/kg body weight dissolved in drinking water daily for 2 months.

**Results:**

When compared with the control group, a statistically significant decrease was demonstrated in the total follicle numbers of the female mice in the nilotinib group (268±110 vs. 170±60; p=0.03). Active spermatogenesis was observed in each tubule sample taken from the mice in the control and nilotinib groups. Spermatogenic activity was similar in the two groups.

**Conclusion:**

We have demonstrated that even though spermatogenesis is preserved, folliculogenesis is inhibited by the usage of a continuous nilotinib treatment dose in chronic myeloid leukemia.

## Abstract (tr)

**Amaç:**

Tirozin kinaz inhibitörlerinin; potansiyel gonadotoksisite ve döllenme üzerindeki olası etkileri hakkında kesin veriler bulunmamaktadır. Bu çalışmanın amacı sağlıklı fare modelinde nilotinib tedavisinin fertilite üzerine etkilerini araştırmaktır.

**Gereç ve Yöntemler:**

On erkek ve on dişi fareye 20 mg/kg/gün nilotinib içme suyuna katılarak 2 ay süre ile verildi.

**Bulgular:**

Folikül sayılarında nilotinib verilen dişi fare grubunda kontrol grubuna göre istatistiksel olarak anlamlı bir azalma olduğu gözlendi (268±110 vs. 170±60; p=0,03). Kontrol ve nilotinib gruplarındaki farelerden alınan her seminifer tübül örneğinde spermatid veya spermatozoa olan aktif spermatogenez gözlendi. Spermatojenik aktivite indeksi her iki grupta da benzerdi.

**Sonuç:**

Bizim çalışmamız ile kronik miyeloid lösemide tedavi dozunda ve uzun süreli nilotinib kullanımı ile spermatogenez korunurken folikülogenezin baskılandığı gösterilmiştir.

## INTRODUCTION

The nilotinib molecule (AMN107) was first described in 2005 by Weisberg et al. [1]. It is a new imatinib-based aminopyrimidine that inhibits BCR-Abl (breakpoint cluster region-Abelson) signalization in the same way that imatinib does [2]. It inhibits not only BCR-Abl, but also other tyrosine kinases such as c-kit, platelet-derived growth factor receptor A/B (PDGFR A/B), Arg (Abelson-related gene), and c-fms (colony-stimulating factor-1 receptor). It is known that those sets of proteins have key roles in gonadal development, implantation, and fetal development [3].

Stem cell factor (SCF)/c-kit is expressed in human ovaries during follicular development, and during inhibition with anti-c-kit antibodies and c-kit receptor antagonists, the number of atretic follicles increases [4]. PDGF protein is also found in the oocytes of primordial and developing follicles, and blocking PDGF decreases the amount of primary follicles. This suggests that PDGF plays a role in the process of transition from a primordial follicle to a primary follicle [4]. However, there are reported cases of healthy deliveries in the literature where one of the parents was undergoing imatinib treatment.

It has been proven that c-kit and its ligand SCF have an effective role in testicular development, migration, proliferation, and survival of germ cells in studies with rodents [5]. Additionally, PDGF is also an important mediator in the maturation of Leydig cells [6]. Therefore, it is thought that inhibition of these developmental signaling pathways might have negative effects on the production of testosterone and normal spermatogenesis [7]. Although androgen levels were decreased in animal studies, pregnancy and live birth can be achieved while male chronic myeloid leukemia (CML) patients are using imatinib [8,9]. Therefore, the reproductive toxicity of nilotinib and other tyrosine kinase inhibitors (TKIs) is still debatable.

On the basis of these findings, we propose that continuous nilotinib treatment may affect folliculogenesis and spermatogenesis in a healthy mouse model. To the best of our knowledge, this is the first identification of nilotinib’s effect on fertility in the mouse and the first study using quantitative measurements of folliculogenesis and spermatogenesis.

## MATERIALS AND METHODS

A total of 40 mice (20 males, 20 females; C57bI6 type, Kobay Animal Experiment Laboratory, Ankara, Turkey) were used in our study, which were 5 weeks old, weighed 20-22 g, and had never copulated before.

During the whole study, experimental subjects were kept in custom-made standard cages made for mice, which had plastic bottoms and sides and wire fencing covering the tops. A group of mice (10 each) were put in each cage. The bottoms of the cages were always kept covered with dry shavings. Shavings were replaced once every 2 days. During the experiment, the mice were kept under optimum laboratory conditions [22±1 °C, 12-h light/dark cycle (07:00/19:00)] and were fed with fabricated pellets containing 21% crude protein, which were specially produced for small experimental animals, and fresh water.

This study was carried out with the permission of the local ethics committee with decision number 67 and date 18.03.2013.

### Experimental Groups

The experimental study comprised 4 groups, each consisting of 10 randomly picked mice:

Group I (n=10): female mice given nilotinib.

Group II (n=10): male mice given nilotinib.

Group III (n=10): control female mice.

Group IV (n=10): control male mice.

Each mouse received 0.4 mg of nilotinib (Tasigna^TM^, Novartis Pharma, Basel, Switzerland) per day dissolved in drinking water for 2 months.

The dose of nilotinib (20 mg/kg, oral) was based on the plasma concentration measured in clinical trials of the dose used today [10]. For this dose, the active ingredient in the 200 mg capsule was dissolved in drinking water, and after making sure that the mixture was homogeneous, 0.4 mL of the mixture was extracted and added to the drinking water of the mice in the nilotinib groups. At the same time, the mice in the control groups (n=10 females and 10 males) received only drinking water.

### Histological Evaluation

At the end of 2 months, mice were sacrificed by cervical dislocation and their ovaries and testicles were processed as described below.

Ovarian tissues were fixed in 10% (w/v) neutral-buffered formalin and embedded in paraffin. Serial sections of 5 µm were then made on a microtome (Leica RM 2125RT, Wetzlar, Germany) for histological evaluation and mounted on glass slides. The slides were stained with hematoxylin and eosin (H&E) (Merck, Darmstadt, Germany) as described below and analyzed for morphological grading and follicle count. Follicles were classified as primordial, primary, or secondary (antral) follicles and primordial follicles were counted for evaluation of ovarian reserve. Follicle numbers in primordial, primary, and secondary phases were recorded in every five sections where the oocyte nucleus was visible in the section, to avoid repetitive evaluations of the same follicle. Follicular atresia incidence was morphologically scored among follicle stages (Table 1). Shrunken oocyte and/or pyknotic granulosa cells were considered as signs of atresia.

For testicles, tissues were fixed in Bouin’s fixative (750 mL of picric acid, 250 mL of 40% formaldehyde, 5 mL of glacial acetic acid) for 2 h at room temperature followed by paraffin embedding and serial sections of 5 µm were made on a microtome (Leica RM 2125RT) for histological evaluation. Spermatogenic scoring and tubule diameter measurements were determined using AxioCam MRc5 software (Carl Zeiss, Göttingen, Germany). The sections were stained with H&E. Briefly, sections were deparaffinized with xylol and rehydrated in decreasing concentrations of alcohol and distilled water before hematoxylin staining for 3 min. Following eosin staining for 1 min, excess eosin was washed off with increasing concentrations of alcohol. Slides were covered with a cover slip using Entellan^®^ (Electron Microscopy Sciences, Hatfield, PA, USA). Twenty-four transversely sectioned seminiferous tubules were assessed under 400^x^ magnification and tubule diameters were measured under 200^x^ magnification.

### Statistical Analysis

Data were analyzed using SPSS 16. The results were given as average ± standard deviation. The t-test was used in evaluation of the relationship between numeric variables. A value of p<0.05 was considered statistically significant.

## RESULTS

### Histological Evaluation of Ovarian Functions

**Figure 1:**
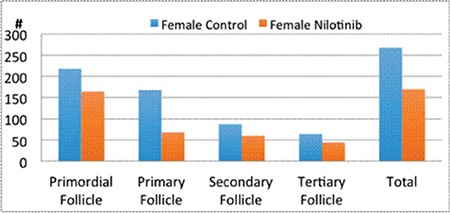
Follicle distributions of female mice in control and nilotinib groups.

One mouse in the nilotinib group was excluded from statistical analysis because of death on the 15^th^ day of the study. When compared with the control group, a statistically significant decrease was observed in the total follicle numbers of the female mice in the nilotinib group (268±110 vs. 170±60; p=0.03). Follicle distributions of mice in the control and nilotinib groups are shown in Figure 1. Although there were no statistically significant differences between the two groups for the numbers of primordial, secondary, and tertiary follicles, the primary follicle numbers in the nilotinib group were significantly lower than in the control group (168±56 vs. 68±25; p=0.02) (Table 2).

**Figure 2:**
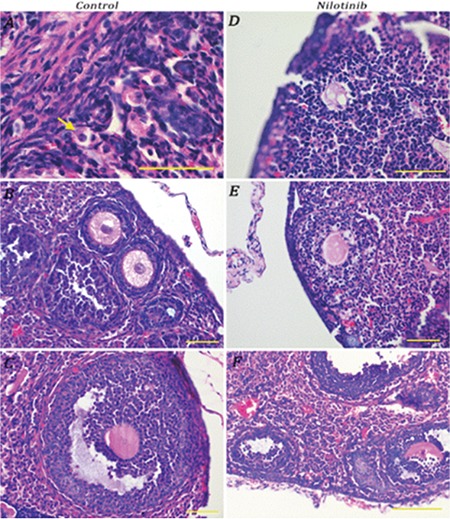
Hematoxylin and eosin-stained ovaries with sections from control [A) primordial (arrow), B) primary, and C) secondary follicles] and nilotinib [D) degenerated primary, E) degenerated primary, and F) degenerated primary and secondary follicles] groups. Bars indicate 50 µm.

In the nilotinib group, ovarian structure was observed to be irregular. It was seen that the gap between the cortex and medulla was narrower than it would normally be. The follicles, which should be organized from the periphery to the medulla in terms of the level of development in their normal hierarchical order, were sparse and scattered in the nilotinib group (Figure 2).

### Histological Evaluation of Testicular Functions

**Figure 3:**
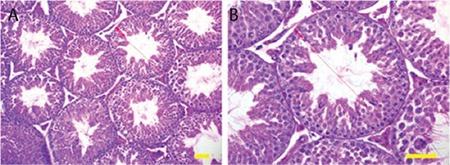
Hematoxylin and eosin-stained sections of nilotinib group testicle (A) and a detailed seminiferous tubule (B). Bars indicate 50 µm.

The morphological appearances of the testicles were similar in the control and nilotinib groups (Figure 3). No pathological symptoms were observed in the tubular or interstitial areas. Active spermatogenesis was observed in each tubule sample taken from the mice in the control and nilotinib groups. Spermatogenic activity was similar in both groups (Table 3; p=0.241). No statistical difference was observed between the mean diameter values of seminiferous tubules in the control and nilotinib groups (Table 3; p=0.475).

## DISCUSSION

To the best of our knowledge, this is the first study that postulated the effects of nilotinib with a continuous CML treatment dose on fertility by quantitative histopathological measurement of spermatogenesis and folliculogenesis in a healthy mouse model. We evaluated the effect of nilotinib on ovarian reserve by the change of follicular number. As expected, the follicle numbers of the female mice were significantly decreased in the nilotinib group compared to the control group (268±110 vs. 170±60; p=0.03). While a statistically significant decrease occurred in the numbers of primary follicles in the nilotinib group (168±56 vs. 68±25; p=0.02), a similar follicular stage was observed between the nilotinib and control groups (p>0.05).

Primordial follicles are the dormant pool of female gametes from which all mature oocytes for ovulation and fertilization originate. There is evidence from organ culture and animal experiments that PDGF and KIT-ligand promote the growth of oocytes and stimulate the transition of primordial follicles to a growing state [11]. Treatment with TKIs might inhibit recruitment of primordial follicles and could lead to irregular menstrual cycles or even amenorrhea [12].

Schultheis et al. [12] reported no effects of tyrosine kinase inhibition with 150 mg/kg/day imatinib for 2 months on folliculogenesis in the ovaries of Bcr-Abl xenografted mice. However, unlike in mice, development of primary ovarian deficiency was reported in the second year of treatment in a 28-year-old female CML patient using imatinib [13].

There is no study in the literature considering the effects of second-generation TKIs on folliculogenesis. The effects on estrous cycle, pregnancy, and copulation were observed in mature female mice after administration of >60 mg/kg/day nilotinib [http://multimediacapsule.thomsonone.com/novartis/nilotinib-tfr-asco]. On the basis of these data, nilotinib does not cause female infertility [14]. However, the clinical data confirming this claim consist of 2 cases [15,16]. In the case report that Conchon et al. [16] published in 2009, a 30-year-old woman with CML using nilotinib had two healthy births once in 2 years. In a single-center retrospective analysis of the pregnancy incidences among female CML patients using TKIs, it was reported that 1 out of 25 pregnancies was achieved under nilotinib and a healthy baby was born [15]. Several cases of successful pregnancies have been reported with TKIs; however, the usage of any TKI during pregnancy must be avoided in light of the present data.

Our study has revealed the effects of nilotinib on spermatogenesis with histopathological parameters. Measuring the seminiferous tubule diameter is a crucial parameter in terms of showing testicular reserves. While the mean seminiferous tubule diameter in the control group was 190.6±8.3 µm, it was measured as 194.3±7.3 µm in the nilotinib group (p=0.475). The spermatogenic activity, which is calculated by grading according to the existence of germ cells, motivic phase, spermatids, and spermatozoa, was similar in both of the groups. Our findings in this mouse model indicate that nilotinib has no effect on spermatogenesis, since there was a normal succession of sperm production in the testes.

PDGFR and KIT-ligand play critical roles in regulating the development and functional control of the testis. TKIs interfere with various maturation processes in animal models, including gonocyte migration, growth of the testis, formation of spermatogonial stem cells, and Leydig cell survival [7].

Nurmio et al. [7] reported that postnatal testicular development was permanently affected in rats when PDGFR and c-kit tyrosine kinases were inhibited by oral imatinib (150 mg/kg/day) during the first postnatal week. The age of the mice might be another explanation for the failure to detect an effect of nilotinib on the testes in our study. It was suggested that some limited phenotypes of mice with single mutations probably indicate different explanations for this redundancy of the tyrosine protein kinase pools in testis mutations [17]. Experiments with large numbers of animals including copulation models are needed.

A decrease of total epididymal weights was reported in adult male mice that were given 180 mg/kg/day nilotinib, and deleterious effects on fertility leading to decrease in the number of sperm and sperm motility have been observed [http://multimediacapsule.thomsonone.com/novartis/nilotinib-tfr-asco]. On the basis of these data, it is currently reported that men taking nilotinib might experience negative effects on fertility [14]. In the literature, oligozoospermia was seen with the imatinib treatment of an 11-year-old male CML patient [6]. However, there are also male CML patients in the literature who had healthy children while taking imatinib. Between the years 2003 and 2014, a total of 109 pregnancies were reported among male CML patients on imatinib and 103 of these pregnancies resulted in healthy babies [8,15,18,19,20,21,22].

The treatment of CML is based on chronic tyrosine kinase inhibition. Imatinib mesylate, which is a Bcr-Abl TKI, has been used since 2001 and has become the cornerstone of CML treatment. In 2006, second-generation TKIs (nilotinib, dasatinib) were put into clinical practice. In the recently published Stop Imatinib Study, it was reported that imatinib treatment might safely be stopped among patients who maintained major molecular response [23]. These positive consequences increased the expectations of many patients of reproductive age who wished to have a child. However, there are no certain data related to the probable effects of TKIs on potential gonadotoxicity and fertility. Among TKIs, only the effect of imatinib was demonstrated, and these data from animal models have been conflicting.

Young patients with hematological malignancies should be advised about the risks of treatment affecting their reproductive potential and the chances of a successful outcome of pregnancy. Cryopreservation of sperm and ovarian tissues or embryos before treatment is a significant option for patients who want to have a child in the future. However, this should be handled cautiously since malignant cells might infiltrate the tissues that are meant to be autotransplanted in the future. It is important to note that ovarian tissue involvement may also be seen in patients with CML [24]. Even testicular involvement was reported in the blastic phase of CML cases [25]. Considering all these possibilities, we preferred to use a healthy mouse model in our study in order to avoid leukemic infiltration; thus, it was possible for us to determine only the effects of the drug on the gonads.

## CONCLUSION

In this study, the effect of nilotinib on spermatogenesis and folliculogenesis in a healthy mouse model was evaluated by using histopathological parameters. We have shown that even though spermatogenesis is preserved, folliculogenesis is inhibited by the usage of a continuous CML treatment dose of nilotinib. There is a need for further randomized and controlled studies with primate and human models to elucidate the effects of nilotinib and endocrinopathies by hormonal measurements, such as estradiol, testosterone, follicle-stimulating hormone, luteinizing hormone, and anti-Müllerian hormone.

## Figures and Tables

**Table 1:**
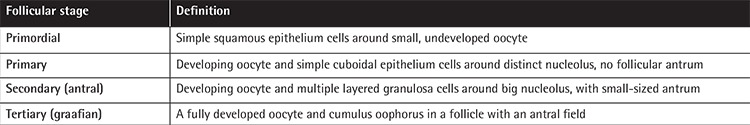
Classification of ovarian follicles [26].

**Table 2:**
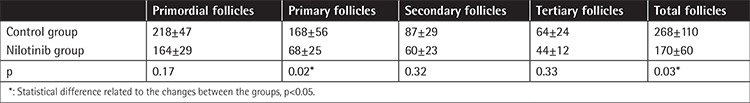
Number of follicles in nilotinib and control groups (mean ± standard deviation).

**Table 3:**
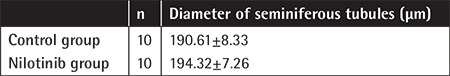
Comparison of spermatogenesis in control and nilotinib groups (mean ± standard deviation); no statistical difference in terms of intergroup changes at p>0.05.
